# Development and External Validation of Nomograms for Predicting Survival in Nasopharyngeal Carcinoma Patients after Definitive Radiotherapy

**DOI:** 10.1038/srep15638

**Published:** 2015-10-26

**Authors:** Lin Yang, Shaodong Hong, Yan Wang, Haiyang Chen, Shaobo Liang, Peijian Peng, Yong Chen

**Affiliations:** 1 Sun Yat-sen University cancer center, 651 Dongfeng Road east, Guangzhou, China; 2 State Key Laboratory of Oncology in Southern China, Guangzhou, China; 3 Collaborative Innovation Center for Cancer Medicine, Guangzhou, China; 4 The Six Affiliated Hospital of Sun Yat-sen University, Guangzhou, China; 5 The First Hospital of Foshan, Foshan, China; 6 The Fifth Affiliated Hospital of Sun Yat-sen University, Zhuhai, China

## Abstract

The distant metastasis free survival (DMFS) and overall survival (OS) differ significantly among individuals even within the same clinical stages. The purpose of this retrospective study was to build nomograms incorporating plasma EBV DNA for predicting DMFS and OS of nasopharyngeal carcinoma (NPC) patients after definitive radiotherapy. A total of 1168 non-metastatic NPC patients from two institutions were included to develop the nomograms. Seven and six independent prognostic factors were identified to build the nomograms for OS and DMFS, respectively. The models were externally validated by a separate cohort of 756 NPC patients from the third institutions. For predicting OS, the c-index of the nomogram was significantly better than that of the TNM staging system (Training cohort, *P* = 0.005; validation cohort, *P* = 0.03). The c-index of nomogram for DMFS in the training and validation set were both higher than that of TNM classification with marginal significance (*P* = 0.048 and *P* = 0.057, respectively). The probability of 1-, 3-, and 5-year OS and DMFS showed optimal agreement between nomogram prediction and actual observation. The proposed stratification of risk groups based on the nomograms allowed significant distinction between Kaplan-Meier curves for survival outcomes. The prognostic nomograms could better stratify patients into different risk groups.

Nasopharyngeal carcinoma (NPC) is one of the most common malignancies of head and neck in the Southeast Asia with an annual incidence of 15–50 cases per 100,000 persons, which is closely related to Epstein-Barr virus (EBV) infection^1^. Radiotherapy or chemoradiotherapy is the main treatment for non-metastatic NPC, achieving a 5-year disease-free survival and overall survival (OS) of 84% and 75%, respectively^2^,^3^. However, NPC has higher tendency for metastatic dissemination than other head and neck cancers^4^. Many patients eventually develop distant metastases after definitive radiotherapy and their OS is very poor^5^ . More intensive follow-up and treatment strategies might be needed for high risk patients. However, accurate prediction for OS and metastasis-free survival (DMFS) remains unavailable.

The seventh edition of the American Joint Committee on Cancer (AJCC) TNM staging system is the most widely used prognostic tool, in which non-metastatic NPC patients were stratified according to tumor size and invasion, and the extent of lymph node involvement. However, prediction of survival is far more complicated than TNM staging. For patients with equivalent TNM classification, there remains apparently heterogeneity of DMFS and OS^6^. Other independent prognostic factors could also significantly contribute to the prediction of clinical outcomes. For example, plasma EBV DNA copy number is closely related to tumor burden and could serve as a useful prognostic factor in NPC patients with different clinical stages^7^. Attempts are also made to reveal the prognostic significance of some laboratory index such as serum C-reactive protein (CRP), lactate dehydrogenase (LDH) and hemoglobulin^8^,^9^ . However, until now no acknowledged and validated prognostic models are available.

The optimal follow-up strategies after definitive radiotherapy for NPC patients are still undefined. Identifying subgroups of patients at different risks for distant metastases could help determine the appropriate timing and imaging techniques in a more individualized manner. Also, more accurate prediction of OS could be of significance for both patients and clinicians in decision making.

Nomograms have been accepted as reliable and pragmatic prediction tools to quantify individual risk by incorporating a variety of important factors for oncological prognoses. In many types of cancers, nomograms have been proved to provide more precise prediction compared with traditional TNM classification^10^. However, nomograms for predicting DMFS and OS after definitive radiotherapy for non-metastatic NPC patients are rare. In this study, we hypothesized that nomograms combing T stage, N stage and objective laboratory index could generate more accurate prediction models for curative NPC patients.

## Patients and Methods

### Training cohort

The training cohort for nomogram development was derived from Sun Yat-sen University Cancer Center and the First Hospital of Foshan between October 2007 and December 2009. The inclusion criteria for the study are as follows: (i) pathological evidence of NPC; (ii) complete baseline clinical information and laboratory data; (iii) patients had received radical radiotherapy and (iv) complete follow-up data. Patients with distant metastasis at presentation were excluded. Ethical approvals were obtained from both institutions through their respective institutional review boards. Inform consent was granted a waiver due to the retrospective nature of the study. The study protocol was designed in accordance with the guidelines outlined in the Declaration of Helsinki and was approved by the Ethics Committee of Sun Yat-sen University Cancer Center and the First Hospital of Foshan, respectively.

A standardized data collection form was designed to retrieve all the relevant information on sociodemographic data (age, gender, smoking history, alcohol exposure, family history of malignant tumors and household registry), baseline laboratory data (plasma EBV DNA copy number, titers of IgA antibodies against EBV capsid antigen (EBV VCA-IgA, EA-IgA), serum calcium, serum magnesium, serum phosphorus, albumin(ALB), globulin (GLB), alanine transaminase(ALT), asinine transaminase(AST), LDH, alkaline phosphatase (ALP), C-reactive protein (CRP), *et al*.), staging data (T stage based on the location, size and extension of the primary tumor; N stage based on the number and location of lymph node metastasis), therapeutic data (radiotherapeutic technic, radiation fractions and dosage, utility of chemotherapy). Clinical stage was classified according to the seventh edition of the AJCC/UICC TNM Staging System.

### Validation cohort

To examine the generalizability of the model, an external validation cohort of 756 consecutive NPC patients who also received definitive radiotherapy were included from the Fifth Affiliated Hospital of Sun Yat-sen University between January 2007 and December 2010. Only patients with non-metastatic disease were included, and all the patients should have sufficient data to score all the variables in the established nomograms. The study protocol was designed in accordance with the guidelines outlined in the Declaration of Helsinki and was approved by the Ethics Committee of the Fifth Affiliated Hospital of Sun Yat-sen University.

### Follow up

Distant metastasis was evaluated by physical examination, nasopharyngoscope, nasopharyngeal and neck magnetic resonance imaging (MRI), chest x-ray and/or CT, abdominal ultrasonography and bone scan every 6 months during the first three years after the completion of radiotherapy and annually thereafter. Survival follow-up was done via direct telecommunication or by referring to the clinic attendance records.

### Statistical analysis

DMFS was defined as the time from definitive radiotherapy to the time of metastases or censored at the date of last follow-up. OS was defined as the time from diagnosis to the date of death from any cause or censored at the date of last follow-up. In the training dataset, survival curves for different variable values were plotted by Kaplan-Meier estimates and compared by using log-rank test. Variables achieving significant level of *P* <0.05 were entered into multivariate analyses via the Cox proportional hazards model with forward stepwise procedures. Independent prognostic factors were determined if they had significant effect in the Cox model (*P* <0.05). Statistical analyses for survival data were performed by using SPSS 19.0 (SPSS, Chicago, IL). Nomograms were formulated to provide visualized risk prediction based on the results of multivariate analyses by R 2.14.1 (http://www.r-project.org) with the survival and rms packages. The nomogram was subjected to 1000 bootstrap resamples for interval validation and external validation to correct the concordance index (c-index) and explain variance for over-optimism. The performance of the nomograms and TNM staging system for prediction survival were measured by c-index, an equivalent variable of the area under curve (AUC) of the receiver operating characteristic curve for censored data. The maximum value of c-index is 1.0 indicating a perfect prediction model while 0.5 indicates a random chance to correctly predict outcome by the model. Comparisons between nomogram models and TNM staging were performed with the rcorrp.cens in Hmisc in R. Calibration of the nomogram for 1-, 3-, and 5-year OS or DMFS were performed by comparing the predicted survival with the observed survival. During external validation of the nomograms, the total points for each patient were calculated according to the established nomograms and then Cox regression was performed using the total points as predictor in the validation cohort. In addition to numerically comparing the discrimination ability by c-index, we also attempted to demonstrate the independent discrimination ability of the nomograms beyond standard TNM classification. By grouping patients evenly into 3 risk groups in the training cohort according to the scores calculated by the nomograms, we determined the cut-off points of risk stratification and investigate its prediction role in different TNM stages with respective Kaplan-Meier survival curves. A two-sided *P* value of <0.05 was deemed significant. Details of R code for running nomograms could be assessed in supplementary information online.

## Results

### Patient characteristics and survival

A total of 1168 and 756 patients from the training and the external validation cohorts were included for analyses. Median follow-up for OS and DMFS in the training cohort were 70.0 months and 68.8 months, whereas the median follow-up for OS and DMFS in the validation dataset were 61.8 months and 60.25 months. Five-year events rates for OS and DMFS in the training cohort were 84.0% and 85.6%, and were 84.6% for OS and 83.5% for DMFS in the validation cohort. Details regarding patient characteristics are shown in Table 1.

### Univariate analysis and multivariate analysis

For OS, the significant inferior prognostic factors included older age, male sex, smoking, elevated LDH, CRP and plasma EBV DNA, decreased albumin, elevated titers of EA-IgA, higher clinical T stage and N stage.

All significant variables were entered into Cox regression model. The results show that the following variables remained independently prognostic: age, gender, LDH, CRP, plasma EBV DNA, T stage and N stage. Likewise, for DMFS, independent prognostic factors were gender, LDH, CRP, plasma EBV DNA, T stage and N stage.

The detailed results of multivariate analyses are shown in Table 2.

### Prognostic nomogram

**Figure 1:**
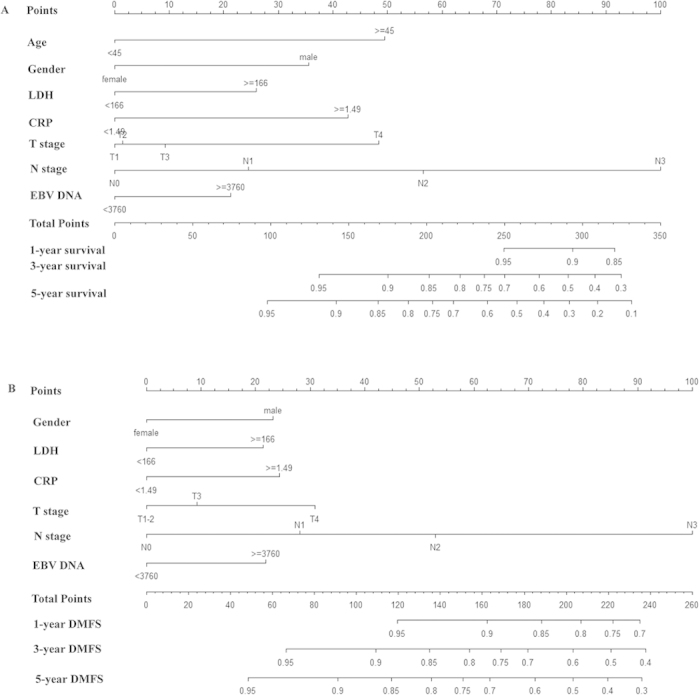
Nomograms of non-metastatic NPC patients after definitive radiotherapy for OS (**A**) and DMFS (**B**). LDH, Lactate dehydrogenase; CRP, C-reactive protein; EBV, Epstein-Barr virus; OS, overall survival; DMFS, disease-metastasis free survival.

The resulting coefficients from the Cox models were used to construct the nomograms for OS and DMFS (Fig. 1). Each subtype within the variables was assigned a score. By adding up the total score from all the variables and locating it to the total point scale, we could determine the probabilities of the outcomes by drawing a vertical line to the total score. N stage was the most important contributing factor both for OS and DMFS prediction. In the training cohort, the nomogram for OS had a bootstrap-corrected c-index of 0.76 (95% CI, 0.73–0.79) which was significantly better than that of TNM classification (0.65; 95% CI, 0.62–0.69; *P* = 0.005). The c-index of nomogram for DMFS (0.71; 95% CI, 0.68–0.75) was also significantly higher than that of TNM classification (0.64; 95% CI, 0.60–0.68; *P* = 0.048). In the external validation cohort, the c-index was 0.74 (95% CI, 0.69–0.78) for OS and 0.69 (95% CI, 0.65–0.74) for DMFS in nomograms, both of which were better than the c-index in TNM classification for OS (0.66; 95% CI, 0.61–0.70; *P* = 0.03) and DMFS (0.62; 95% CI, 0.57–0.66; *P* = 0.057), respectively. The results were shown in Table 3.

**Figure 2:**
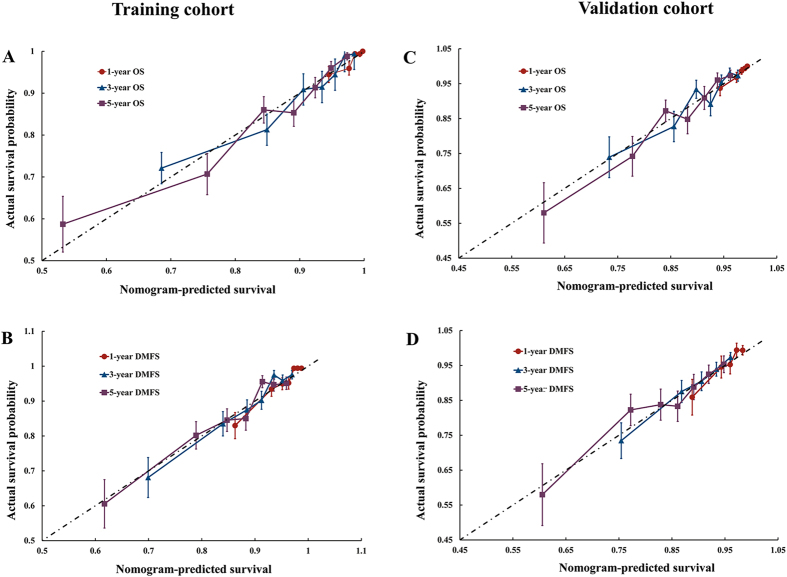
Calibration plots of OS at 1, 3, 5 years (**A**,**C**) and DMFS at 1, 3, 5 year (**B**,**D**) in training cohort (left) and validation cohort (right). Nomogram-predicted OS and DMFS are plotted on the x-axis; actual OS and DMFS are plotted on the y-axis. Dashed lines along the 45-degree line through the origin point represent the perfect calibration models in which the predicted probabilities are identical to the actual probabilities. OS, overall survival; DMFS, disease-metastasis free survival.

The calibration plots presented fair agreements between the nomogram prediction and actual observation for the 1-, 3-, and 5-year OS and excellent agreements for the 1-, 3-, and 5-year DMFS in both cohorts (Fig. 2).

### The nomogram in stratifying risk of patients

**Figure 3:**
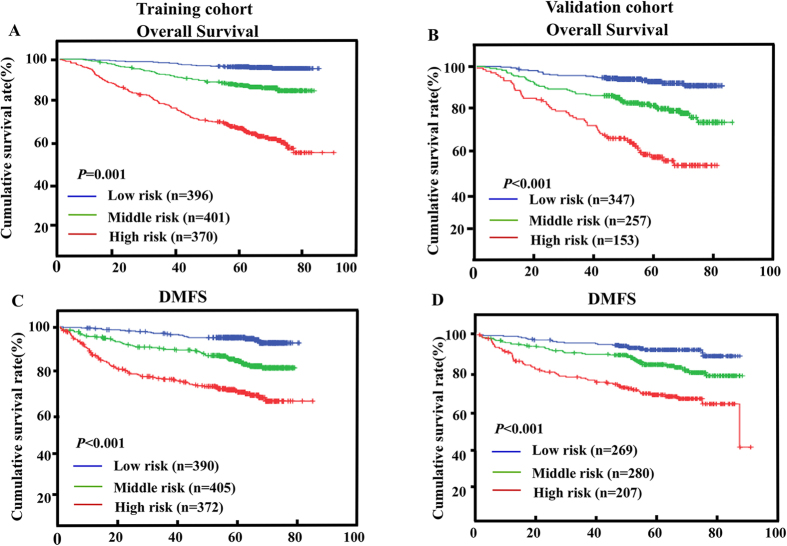
Kaplan–Meier curves of risk group stratification for OS (**A**,**B**) and DMFS (**C**,**D**). Nomogram risk group stratifications for the 33 and 66 percentiles are shown for the training cohort (left) and for the validation cohort (right). OS, overall survival; DMFS, disease-metastasis free survival.

We determined the cutoff values of nomogram-generated scores with which patients in the training cohort were evenly stratified into three risk groups. Each group represented a distinct prognosis (Table 4). This stratification could effectively discriminate the survival outcomes for the three proposed risk groups both in the training and validation cohorts (Fig. 3). Even within different TNM stages, the stratification could allow significant distinction among Kaplan-Meier curves for OS (see Supplementary Figure S1 online) and DMFS (see Supplementary Figure S2 online).

## Discussion

The prediction of survival by TNM staging in NPC patients remains imperfect due to its simplicity and the heterogeneity of risk within the same stage. Developing prediction models with more precision that incorporate a variety of independent objective variables are urgently needed. To the best of our knowledge, this is one of the few studies to develop visualized, user-friendly and reliable prediction models for OS and DMFS in non-metastatic NPC patients based on large database. The nomograms we established showed superior discrimination ability compared with traditional TNM staging and allowed risk scoring for individual patient.

Previously, Cho *et al*. has developed two nomograms to predict the probability of complete response to radiotherapy and OS in non-metastastic NPC patients in Korea, respectively^11^. However, the relatively small sample size and limited number of prognostic factors in this study call for larger studies for more accurate risk prediction. Also, establishing a prediction model for risk of distant metastasis is of clinical significance, which could help clincians develop more individulized multidisciplinary treatment and follow-up strategies for NPC patients.

In the present study, the training cohort was obtained from two institutions in Southern China, both of which is located in the areas with high prevalence of NPC and receives a vast amount of NPC patients each year, guaranteeing the representativeness of NPC in endemic areas. Multivariable analyses show that variables include age, gender, LDH, CRP, plasma EBV DNA, T stage, and N stage could independently predict OS. For DMFS, the independent prognostic factors were the same with that for OS except that age did not remain significant. Based on the results of Cox regression model, we established the respective nomograms for predicting OS and DMFS with excellent discrimination ability (c-index for OS, 0.76; c-index for DMFS, 0.71). The established nomograms were found to override traditional TNM staging system in predicting OS and DMFS. More importantly, calibration curves show optimal agreements between prediction and actual observation of the studied outcomes, which guarantees the reliability of the established nomograms.

The nomogram models were further validated in an external cohort (*N* = 756) from a third institution to avoid over-fitting of the models and determine their applicability. The c-index of the nomogram predictions for OS and DMFS in the validation cohort were 0.74 and 0.69, respectively, both of which were higher than that of TNM staging predictions. However, the superiority of the discrimination ability of nomogram over TNM classification for predicting DMFS in the validation cohort only had marginal significance (*P* = 0.057). We think this phenomenon could be attributed to the relatively small sample size in the validation cohort or the existence of other potential predictors for distant metastasis, which deserves further investigation in the future.

Based on the nomograms, we further stratify patients into three distinct risk groups for OS and DMFS. Surprisingly, even within the same TNM category, the proposed risk groups could still significantly discriminate the survival outcomes except for the discrimination of OS in the early stage NPC in the validation cohort (*P* = 0.636). One feasible contributor to this insignificance is the small sample size of patients with early stage in this cohort. All in all, these results imply that the nomograms could provide satisfactory discrimination ability beyond TNM staging systems and might be good substitutes for traditional TNM classification.

Age at diagnosis was a strong prognostic factor for OS. Increasing age adversely affected OS in NPC patients after definitive radiotherapy^12^. The increased risk of comorbidities, less tolerance to intensive therapies and the declined immune function with increasing age might account for the inferior OS in the elder patients^13^. This results call for the involvement of multidisciplinary approaches apart from oncological aspects to further improve the outcome of elderly patients. Also, clinical trials with special attention to older patients are warranted.

We also found that females had better prognoses than male patients. This phenomenon might be explained by the fact that testosterone could negatively affect the immune function in men, whereas female hormones might have an immune enhancing role^14^. However, the mechanisms underlying gender differences in the prognosis of NPC could not be fully demonstrated unless solid biological studies are available.

The link between inflammation and cancer is well-established^15^. CRP is a non-specific, acute phase marker of inflammation which has been proved to be associated with inferior survival of numerous malignancies^16^,^17^. In this study we also found that CRP has moderate contribution to the nomogram prediction of OS and DMFS. In the future, manipulating the inflammatory status and the immune function of NPC patients might be a promising strategy to further improve their clinical outcomes.

In the present study, we further confirmed that elevated serum LDH is inversely related to patients’ survival. Consistently, another retrospective study showed that high pretreatment LDH was correlated with poorer 4-year OS and DMFS in NPC^9^. The link between LDH and survival might rely on cancer hypoxia^18^. Hypoxia is a characteristic property of NPC due to rapid cell proliferation, high metabolic demands and impaired angiogenesis. In response to hypoxic stress, anaerobic glycolysis becomes the main energy source for cancer cells, resulting in up-regulation of LDH and activation of the hypoxia-inducible factor (HIF) pathway and ultimately impairment of immune response and survival^19^.

EBV infection is associated with increased risk for NPC in endemic areas^20^. Several studies have substantiates that circulating EBV DNA originates from the EBV-infected primary tumor cells which reflect the overall tumor load and tumor metabolic activity^21^. Plasma EBV DNA level (either prior to treatment or during follow-up) has been proved to be a significant marker in the prognostication of both non-disseminated and disseminated NPC^22^. However, most studies simply investigated the prognostic value of plasma EBV DNA as a single variable and lack solid validation, whereas the present study for the first time incorporated plasma EBV DNA into prognostic models and achieved more accurate discrimination of survival in NPC patients, further confirming the prognostic value of plasma EBV DNA in NPC patients after curative radiotherapy.

Of course, our nomograms have some limitations. First, the nomograms only include basic clinical and laboratory data. However, the present study aimed to build reliable prediction models. Objective variables are therefore the most ideal factors to be included, while subjective variables might negatively impact the models due to inevitable bias. Second, the study was conducted retrospectively and selection bias might exist. However, we have included a relatively large training cohort to build the nomograms and externally validated them. The results consistently show the satisfactory performance of the established models. Of course, additional validation of these nomograms by prospective datasets could be useful. Overall, the established nomograms predicting OS and DMFS in NPC patients after definitive radiotherapy provide practical tools for individualized prognostication. Practitioners as well as patients could readily assess these nomograms and immediately apply them to predicting the risks of distant metastasis and death. By doing this, tailored post-treatment follow-up and/or adjuvant therapy could be feasible and improved survival might be achieved.

## Additional Information

**How to cite this article**: Yang, L. *et al*. Development and External Validation of Nomograms for Predicting Survival in Nasopharyngeal Carcinoma Patients after Definitive Radiotherapy. *Sci. Rep*. **5**, 15638; doi: 10.1038/srep15638 (2015).

## Supplementary Material

Supplementary Information

## Figures and Tables

**Table 1:** Clinical and laboratorial characteristics of patient.

Characteristics	Training set			Testing set
	Number of cases (%)	Univariable		Number of cases (%)
		OS	DMFS	
Age(years)		<0.001	0.189	
<45	602(51.5%)			404(53.4%)
≥45	566(48.5%)			352(46.6%)
Gender		<0.001	0.037	
Male	853(73%)			555(73.4%)
Female	315(27%)			201(26.6%)
Smoking status		<0.001	0.008	
Absent	705(60.4%)			498(65.9%)
Present	463(39.6%)			258(34.1%)
Drinking status		0.211	0.183	
Absent	971(83.1%)			526(69.6%)
Present	197(16.9)			230(30.4%)
Family history		0.254	0.273	
Absent	842(72.1%)			576(76.2%)
present	326(27.9%)			180(23.8%)
T classification		<0.001	0.001	
1+2	376(32.2%)			240(31.7%)
2	547(46.8%)			358(47.4%)
3	245(21.0%)			158(20.9%)
N classification		<0.001	<0.001	
0	246(21.1%)			162(21.4%)
1	425(36.4%)			305(40.3%)
2	310(26.5%)			201(26.6%)
3	187(16.0%)			88(11.6%)
Clinical Stage				
I	29(2.5%)			28(3.7%)
II	199(17%)			120(15.9%)
III	620(53.1%)			413(54.6%)
IV	320(27.4%)			195(25.8%)
Treatment method				
RT	221(18.9%)			182(24.1%)
Chemo-RT	947(81.1%)			574(75.9%)
Radiotherapy technology				
IMRT+3DCRT	496(42.5%)			498(65.9%)
CRT	672(57.5%)			258(34.1%)
Radiation fractions				
<=34	602(51.5%)			356(47.1%)
>34	566(48.5%)			400(52.9%)
Radiation dosage(Gy)				
<=69	561(48.0%)			392(51.9%)
>69	607(52.0%)			364(48.1%)
Calcium		0.582	0.473	
<2.46	554(47.4%)			380(50.3%)
>=2.46	614(52.6%)			376(49.7%)
Magnesium		0.924	0.517	
<0.9	633(54.2%)			398(52.6%)
>=0.9	535(45.8%)			358(47.4%)
Phosphorus		0.572	0.989	
<1.15	563(48.2%)			374(49.5%)
>=1.15	605(51.8%)			382(50.5%)
WBC, ×10^9^		0.188	0.302	
<6.9	608(52.1%)			413(54.6%)
>=6.9	560(47.9%)			343(45.4%)
Neutrophil, ×109		0.787	0.804	
<4.1	601(51.5%)			400(52.9%)
>=4.1	567(48.5%)			356(47.1%)
Neutrophil/WBC		0.714	0.724	
<0.61	611(52.3%)			413(54.6%)
>=0.61	557(47.7%)			343(45.4%)
HGB, g/L		0.339	0.686	
<143	596(51%)			369(48.8%)
>=143	572(49%)			387(51.2%)
GLB, g/L		0.661	0.524	
<29	594(50.9%)			380(50.3%)
>=29	574(49.1%)			376(49.7%)
ALB, g/L		0.001	0.371	
<45.6	585(50.6%)			350(46.3%)
>=45.6	583(49.9%)			406(53.7%)
ALT, U/L		0.635	0.135	
<20.6	585(50.1%)			358(47.4%)
>=20.6	583(49.9%)			398(52.6%)
AST, U/L		0.054	0.132	
<20.8	588(50.3%)			377(49.9%)
>=20.8	580(49.7%)			379(50.1%)
ALP, U/L		0.062	0.382	
<66.7	591(50.6%)			373(49.3%)
>=66.7	577(49.4%)			383(50.7%)
LDH, U/L		<0.001	<0.001	
<166	583(49.9%)			379(50.1%)
>=166	585(50.1%)			377(49.9%)
CRP, mg/L		<0.001	<0.001	
<1.49	583(49.9%)			381(50.4%)
>=1.49	585(50.1%)			375(49.6%)
EBV-DNA, copies/ml		<0.001	<0.001	
<3,760	614(52.6%)			453(59.9%)
>=3760	554(47.4%)			303(40.1%)
VCA-IgA		0.657	0.368	
<1:320	591(56.5%)			379(50.1%)
>=1:320	455(43.5%)			377(49.9%)
EA-IgA		<0.001	0.300	
<=1:20	664(56.8%)			382(50.5%)
>1:20	504(25.8%)			374(49.5%)
Distant metastasis				
Absent	981(84%)			627(82.9%)
Present	187(16.0%)			129(17.1%)
Living Status				
Live	952(81.5%)			632(83.6%)
Dead	216(18.5)			124(16.4%)

Abbreviations: RT, radiotherapy; Chemo-RT, chemoradiotherapy; CRT, conventional radiotherapy: IMRT, intensity modulated radiation therapy; 3D-CRT, three dimensional conformal radiation therapy; WBC, White cell; HGB, hemoglobin; GLB, Globulin; ALB, Albumin; ALT, Alanine transaminase; AST, Asanine transaminase; ALP, Alkaline phosphatase; LDH, Lactate dehydrogenase; CRP, C-rective protein; EBV-DNA, Epstein-barr virus DNA; OS, overall survival; DMFS, disease metastasis free survival.

**Table 2:** Selected Factors for building the model.

Characteristics	OS			DMFS		
	*P*	95% CI	Hazard Ratio	*P*	95% CI	Hazard Ratio
Age(year)	<0.001	1.679–2.988	2.240			
<45						
>=45						
gender	0.001	0.392–0.801	0.560	0.030	0.476–0.964	0.677
Male						
Female`						
LDH, U/L	0.003	1.150–2.207	1.527	0.018	1.065–1.930	1.433
<166						
>=166						
CRP, mg/L	<0.001	1.484–2.706	2.004	0.008	1.113–2.039	1.506
<1.49						
>=1.49						
EBV-DNA, copies/ml	0.017	1.063–1.878	1.413	0.018	1.065–1.958	1.444
<3,760						
>=3760						
T classification	<0.001			0.028		
1+2						
3	0.470	0.799–1.627	1.140	0.401	0.812–1.682	1.169
4	<0.001	1.491–3.124	2.159	0.011	1.124–2.509	1.679
N classification	<0.001			<0.001		
0						
1	0.110	0.913–2.440	1.492	0.085	0.937–2.750	1.650
2	<0.001	1.546–4.093	2.516	0.001	1.424–4.177	2.439
3	<0.001	3.122–8.319	5.096	<0.001	3.152–9.168	5.376

Abbreviations: LDH, Lactate dehydrogenase; CRP, C-rective protein; EBV-DNA, Epstein-barr virus DNA; OS, overall survival; DMFS, disease-metastasis free survival.

**Table 3:** The c-index of OS and DMFS for multivariate model performance and TNM in the training set and the validation set.

Model for Survival prediction	Training			Validation		
	C-index	95% CI	*P*	C-index	95% CI	*P*
Nomogram(OS)	0.76	0.73–0.79	0.005	0.74	0.69–0.78	0.03
TNM classification(OS)	0.65	0.62–0.69		0.66	.0.61–0.70	
Nomogram(DMFS)	0.71	0.68–0.75	0.048	0.69	0.65–0.74	0.057
T TNM classification(DMFS)	0.64	0.60–0.68		0.62	0.57–0.66	

Abbreviations: OS, overall survival; DMFS, disease-metastasis free survival.

**Table 4:** Point Assignment from nomograms and Prognostic Score.

Characteristics	OS		DMFS	
	Score	Estimated 5-year OS	Score	Estimated 5-year DMFS
Age(year)				
<45	0			
>=45	50			
Gender				
Male	36		23	
Female`	0		0	
LDH, U/L				
<166	0		0	
>=166	26		21	
CRP, mg/L				
<1.49	0		0	
>=1.49	43		24	
EBV-DNA, copies/ml				
<3,760	0		0	
>=3760	21		22	
T classification				
1+2	0		0	
3	8		9	
4	47		31	
N classification				
0	0		0	
1	25		28	
2	57		53	
3	100		100	
Total prognostic score				
Training cohort				
Low risk	<119	96.2	<80	95.3
Middle risk	119–177	87.7	80–122	87.4
High risk	>177	66.8	>122	73.0
Validation cohort				
Low risk	<119	94.3	<80	92.4
Middle risk	119–17	84.4	80–12	85.0
High risk	>177	69.5	>122	69.8

Abbreviations: LDH, Lactate dehydrogenase; CRP, C-rective protein; EBV-DNA, Epstein-barr virus DNA; OS, overall survival; DMFS, disease-metastasis free survival.
