# Supplementary material for: Development and External Validation of Nomograms for Predicting Survival in Nasopharyngeal Carcinoma Patients after Definitive Radiotherapy
Source: Sci Rep. 2015 Oct 26;5:15638. doi: 10.1038/srep15638 (PMC4620487; doi:10.1038/srep15638)
Supplement: Supplementary Information [file srep15638-s1.pdf]

Supplementary Information

Development and External Validation of Nomograms for Predicting Survival in Nasopharyngeal Carcinoma Patients after Definitive Radiotherapy

Lin Yang[1,2,3#], Shaodong Hong[1,2,3#], Yan Wang[1,2,3#], Haiyang Chen[4], Shaobo Liang[5], Peijian Peng[6], Yong Chen[1, 2,3]*

Affiliations

1. Sun Yat-sen University Cancer Center, 651 Dongfeng Road East, Guangzhou, China
2. State Key Laboratory of Oncology in Southern China, Guangzhou, China
3. Collaborative Innovation Center for Cancer Medicine, Guangzhou, China
4. The Six Affiliated Hospital of Sun Yat-sen University, Guangzhou, China
5. The First Hospital of Foshan, Foshan, China
6. The Fifth Affiliated Hospital of Sun Yat-sen University, Zhuhai, China

Corresponding Author:

*Prof Yong Chen, Sun Yat-sen University cancer center, 651 Dongfeng Road east, Guangzhou 510060, China, 0086-020-87343505, (E-mail: chenyong@sysucc.org.cn)

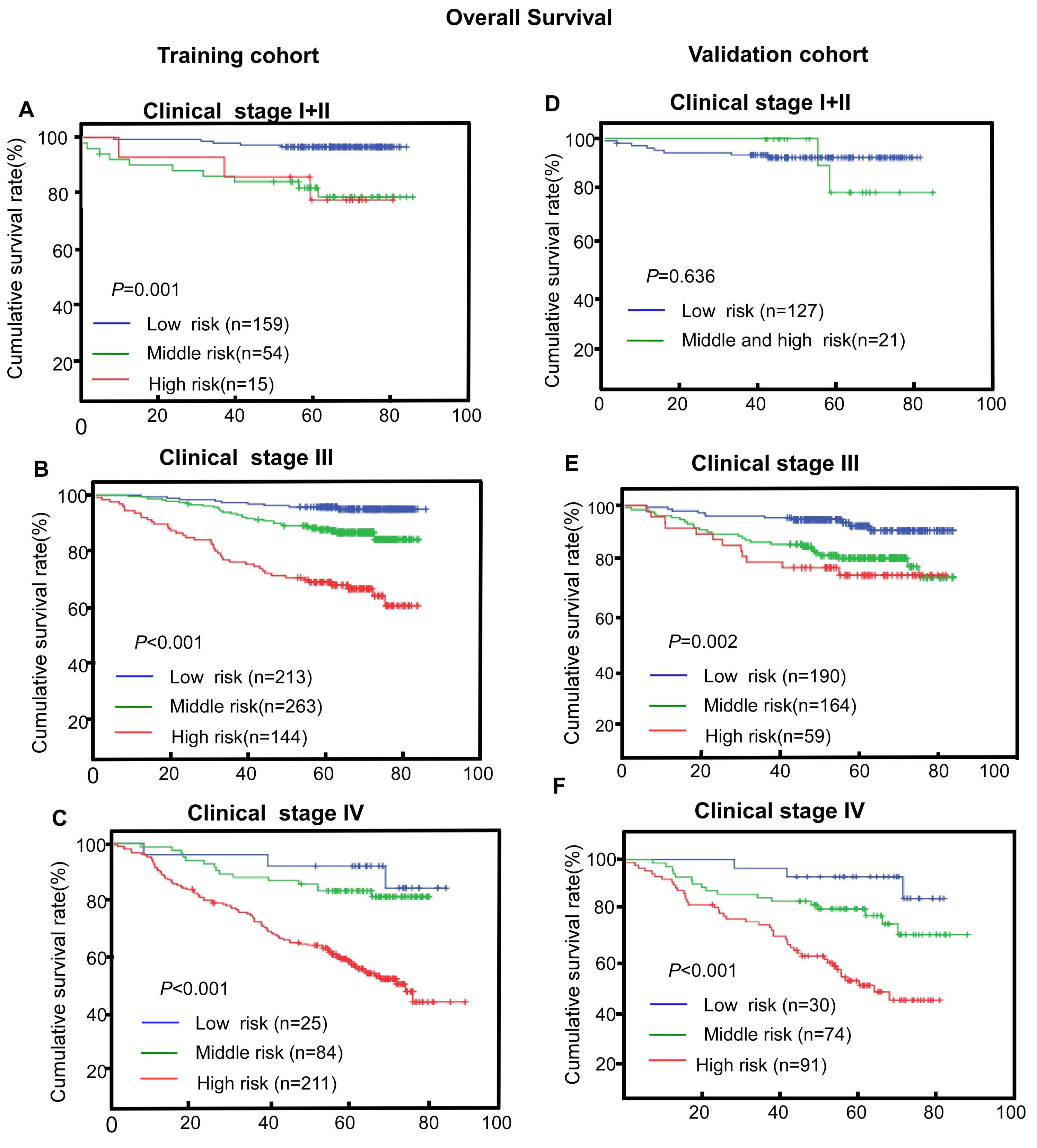

Supplementary Figure S1. Risk group stratification for OS within each TNM stage in the training cohort (left), and for the validation cohort (right). OS, overall survival.

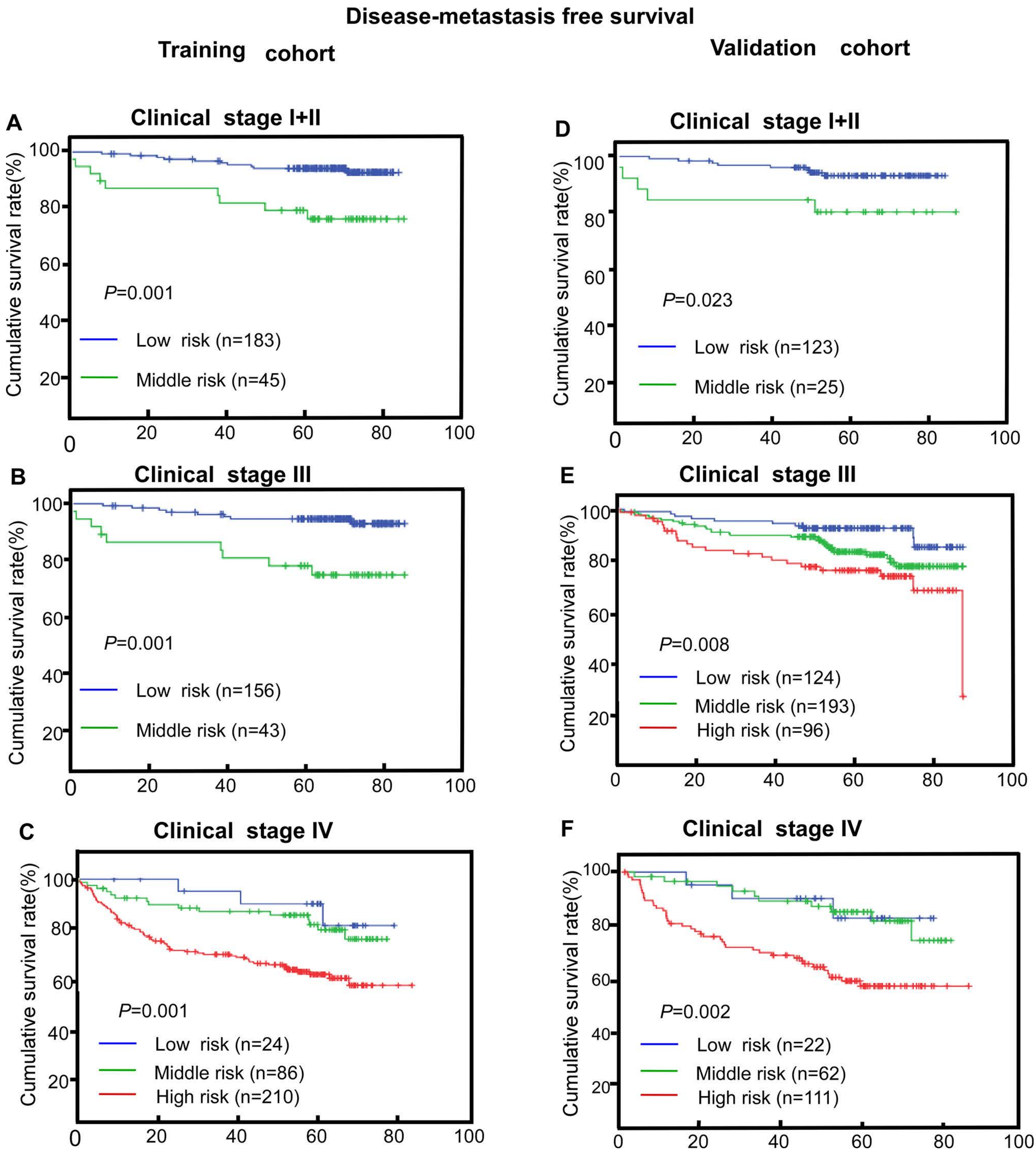

Supplementary Figure S2. Risk group stratification for DMFS within each TNM stage in the training cohort (left) and for the validation cohort (right). DMFS, disease-metastasis free survival.

R code for nomogram construction and validation

This is the R code of nomogram development and validation of the primary cohort using R software. (The validation was conducted by the biostatisticians using the same methods.)

#construction of nomograms

```
setwd("D:/")
library(MASS)
library(foreign)
library(splines)
library(rms)
lc<-read.spss("NPC_training.sav",use.value.labels=T,to.data.frame=T)
attach(lc)
coxm<-cph(Surv(OS,OSstatus)~age+gender+LDH+CRP+Tstage+Nstage+EBV,x=T,y
=T,data=lc,surv=T)
scoxm<-step(coxm)
dd<-datadist(lc)
options(datadist="dd")
surv<-Survival(scoxm)
surv1<-function(x) surv(1*365,lp=x)
surv2<-function(x) surv(3*365,lp=x)
surv3<-function(x) surv(5*365,lp=x)
nom <-nomogram(scoxm,fun=list(surv1,surv2,surv3),lp=F,funlabel=c('1-year
survival','3-year survival','5-year survival'),maxscale=100,fun.at=c
(1.00,0.95,0.9,0.85,0.8,0.75,0.7,0.6,0.5,0.4,0.3,0.2,0.1,0))
plot(nom, xfrac=.45)
print(nom)
```

#resampling internal validation

```
set.seed(1)
validate(coxm, B=1000, dxy=TRUE)
```

#calibration @1 years OS

```
coxm<-cph(Surv(OS,OSstatus)~age+gender+LDH+CRP+Tstage+Nstage+EBV,x=T,y
=T,data=lc,surv=T,time.inc=365)
cal<-calibrate(coxm, cmethod='KM', method='boot',u=365,B=10)
cal
plot(cal, errbar.col = c(rgb(0, 255, 0, maxColorValue = 255)),col = c(rgb(0, 124, 194,
maxColorValue = 255)))
box(lwd = 2)
```

R code for nomogram construction and validation-continued

```
abline(0, 1, lty =4, lwd = 2, col = c(rgb(0, 0, 0, maxColorValue = 255)))
```

# calibration @3 years OS

```
coxm<-cph(Surv(OS,OSstatus)~age+gender+LDH+CRP+Tstage+Nstage+EBV,x=T,y
=T,data=lc,surv=T,time.inc=1095)
cal<-calibrate(coxm, cmethod='KM', method='boot',u=1095,B=10)
cal
plot(cal, errbar.col = c(rgb(0, 255, 0, maxColorValue = 255)),col = c(rgb(0, 124, 194,
maxColorValue = 255)))
box(lwd = 2)
abline(0, 1, lty =4, lwd = 2, col = c(rgb(0, 0, 0, maxColorValue = 255)))
```

# calibration @5 years OS

```
coxm<-cph(Surv(OS,OSstatus)~age+gender+LDH+CRP+Tstage+Nstage+EBV,x=T,y
=T,data=lc,surv=T,time.inc=1825)
cal<-calibrate(coxm, cmethod='KM', method='boot',u=1825,B=10)
cal
plot(cal, errbar.col = c(rgb(0, 255, 0, maxColorValue = 255)),col = c(rgb(0, 124, 194,
maxColorValue = 255)))
box(lwd = 2)
abline(0, 1, lty =4, lwd = 2, col = c(rgb(0, 0, 0, maxColorValue = 255)))
```

#calculation of c-index

```
f<-predict(coxm)
x=rcorr.cens(f,Surv(OS,OSstatus))
se <- x["S.D."]/2
Low95 <- 1-x["C Index"] - 1.96*se
Upper95 <- 1-x["C Index"] + 1.96*se
cbind(1-x["C Index"], Low95, Upper95)
```

#calculation of c-index for TNM staging

```
x3<- rcorr.cens(stage,Surv(OS,OSstatus))
se <- x3["S.D."]/2
Low95 <- 1-x3["C Index"] - 1.96*se
Upper95 <- 1-x3["C Index"] + 1.96*se
cbind(1-x3["C Index"], Low95, Upper95)
```

#comparison of c-index between different models

```
rcorrp.cens(x1, x2, S, Surv(OS, Status))
```
